# Three-dimensional analysis of pancreatic fat by fat-water magnetic resonance imaging provides detailed characterization of pancreatic steatosis with improved reproducibility

**DOI:** 10.1371/journal.pone.0224921

**Published:** 2019-12-02

**Authors:** Shingo Kato, Akito Iwasaki, Yusuke Kurita, Jun Arimoto, Toh Yamamoto, Sho Hasegawa, Takamitsu Sato, Kento Imajo, Kunihiro Hosono, Noritoshi Kobayashi, Masato Yoneda, Takuma Higurashi, Kensuke Kubota, Daisuke Utsunomiya, Atsushi Nakajima

**Affiliations:** 1 Department of Gastroenterology and Hepatology, Yokohama City University School of Medicine, Yokohama, Japan; 2 Department of Oncology, Yokohama City University Hospital, Yokohama, Japan; 3 Diagnostic Radiology, Yokohama City University Graduate School of Medicine, Yokohama, Japan; University College London, UNITED KINGDOM

## Abstract

**Background:**

Since pancreatic steatosis is reported as a possible risk factor for pancreatic cancer, the development of a non-invasive method to quantify pancreatic steatosis is needed. Proton density fat fraction (PDFF) measurement is a magnetic resonance imaging (MRI) based method for quantitatively assessing the steatosis of a region of interest (ROI). Although it is commonly used for quantification of hepatic steatosis, pancreatic PDFF can greatly vary depending on the ROI’s location because of the patchy nature of pancreatic fat accumulation. In this study, we attempted to quantify pancreatic steatosis by fat-water MRI with improved reproducibility.

**Methods:**

Using the MRI images of 159 patients with nonalcoholic fatty liver disease, we attempted to calculate the average PDFF of whole pancreas. We set ROIs covering the entire area of the pancreas appearing in every slice and calculated the average PDFF from all the voxels included in the pancreas. We named this average value as whole-pancreatic PDFF and evaluated the reproducibility of the measured values. In addition to whole-pancreatic PDFF, we measured the average PDFF of the pancreatic head (head-PDFF) and that of the pancreatic body plus tail separately and analyzed their correlation with the clinical characteristics of the patients.

**Results:**

The mean inter-examiner coefficient of variation of the whole-pancreatic PDFF was 11.39%. The whole-pancreatic PDFF was correlated with age (*p* = 0.039), body mass index (*p* = 0.0093) and presence/absence of diabetes (*p* = 0.0055). The serum level of low-density lipoprotein cholesterol was inversely correlated with the head-PDFF.

**Conclusion:**

We developed a new measurement method of the pancreatic PDFF with greater reproducibility. Using this method, we characterized pancreatic steatosis in detail. This novel measurement method allows accurate estimation of the severity of pancreatic steatosis and is therefore useful for the detailed characterization of pancreatic steatosis.

## Introduction

Recently, pancreatic steatosis is reported as a possible risk factor for pancreatic cancer [1, 2], suggesting that the development of a method to quantify pancreatic steatosis may help in early detection of pancreatic cancer. Since pancreatic tissue is difficult to obtain by biopsy, pancreatic steatosis could initially be studied only in autopsy samples; historical analysis revealed a significant correlation between the severity of pancreatic steatosis and the degree of obesity [3], age [3] and presence/absence of type 2 diabetes mellitus (hereinafter called “diabetes”) [3, 4]. However, there is no established method for non-invasive quantification of pancreatic steatosis.

Fat-water magnetic resonance imaging (MRI) is a useful tool for noninvasive fat quantification. Proton density fat fraction (PDFF) calculation is a chemical shift–based water and fat separation technique. Based on T1 relaxation and chemical-shift properties, it can separate fat *F* and water *W* components, and differentiate fat from lean tissues. When regions of interest (ROIs) are set in the organ of interest, the software computes fat percentage in the ROIs on a voxel-by-voxel basis. This fat percentage called PDFF is calculated by following formula; *PDFF* = *F* / (*F* + *W*) x 100% [5].

In the field of research on fatty liver, PDFF is commonly used for non-invasive quantification of hepatic steatosis. We previously demonstrated that MRI based hepatic PDFF has higher diagnostic performance in noninvasive detection of liver steatosis in patients with nonalcoholic fatty liver disease (NAFLD) than transient elastography based fat quantification method [6]. When measuring hepatic PDFF, multiple small ROIs are set in the liver, and the mean PDFF of those ROIs is defined as the sample’s PDFF.

Several groups have applied this MRI-based PDFF measurement method to evaluate the fat percentage of the pancreas [7–11]. In most of these studies, the authors have used a measurement method similar to that used for measuring the hepatic PDFF wherein the mean PDFF is calculated from multiple small ROIs set in the organ. However, this measurement method has not been validated for the measurement of the PDFF in organs other than the liver [10]. Especially in the case of the pancreas, since the fat accumulation is known to be patchy [12], the PDFF values differ widely depending on the locations of the ROIs set in the organ. Therefore, considering the uneven distribution of the patchy nature of pancreatic fat accumulation in pancreatic steatosis, the pancreatic PDFF should ideally not be calculated from parts of the organ, but for the whole organ.

Based on the above background, we attempted to develop a new measuring method for pancreatic PDFF using previously obtained MRI data of NAFLD patients. We performed a three-dimensional analysis of pancreatic fat and calculated average FF from all the voxels included in the pancreas.

## Materials and methods

### Subject characteristics

The study was conducted with the approval of the Ethics Committee of our institution, Yokohama City University in Japan (authorization number: B151001016). In this retrospective observational study, we analyzed the previously obtained MRI images taken to measure hepatic steatosis and clinical data of a total of 159 patients with NAFLD. All the patients underwent liver biopsy between July 2013 and November 2015 at our institution. The histological evaluation method has been described previously [6]. The clinical scoring, MRI and liver biopsy were all performed within 6 months. Patients with a history of excessive alcohol consumption (weekly consumption >140 g for men and >70 g for women), other liver diseases such as chronic hepatitis, history of intake of drugs associated with fatty liver, weight loss, renal diseases, and/or thyroid disorders were excluded from the study. Regarding accessing patient information for this retrospective study, the ethics committee waived the requirement for informed consent. Through the institution's website, we announced that the opportunity to opt-out is always available to patients.

### Assessment of steatosis by MRI-based measurement of the proton density fat fraction

MR examinations were performed with a 3-T imager (GE Healthcare, Milwaukee, WI). The PDFF was measured using the Dixon method with advanced processing, called IDEAL-IQ (Iterative decomposition of water and fat with echo asymmetry and the least squares estimation quantification sequence, GE Healthcare). The Dixon method using 6 echoes was employed to measure the fat fraction using the following imaging parameters: repetition time/echo space: 6.6 ms/0.78 ms; maximum TE 4.8; minimum TE 0.9; NEX 0.50; location per slab 28; number of shots 2; echo train length 3; flip angle: 3 degrees; matrix: 160 x 160; axial imaging plane; section thickness: 8 mm; FOV: 44 cm; phase FOV: 0.8 cm; bandwidth: 111.11 kHz; imaging time, single breath-hold (19 seconds). After setting the ROIs in the organs of interest, the PDFF calculation was performed by a hybrid multipoint water-fat separation approach, where a T2*-corrected, spectrally modeled ‘‘complex based” water-fat separation method (T2*-IDEAL) is followed by a fitting algorithm based on magnitude multi-echo signals as reported previously [13].

### Evaluation of the variations of the hepatic or pancreatic PDFF values depending on the positions of the ROIs

Multiple circular ROIs were drawn in the liver and pancreas. The area of each of the ROIs in the liver was 200 mm^2^, while that in the pancreas was 100 mm^2^. The size of the ROIs for the pancreas was determined regarding previous reports [7–11]. Hepatic PDFF was measured in each of the ROIs in the left and right hepatic lobes. Pancreatic PDFF was measured in each of the ROIs in the pancreatic head, body and tail. The boundary between the pancreatic head and the body was defined by the left edge of the portal vein. The boundary between the pancreatic body and the tail was defined by a line bisecting the caudal pancreas, excluding the head. Ten ROIs were drawn in each area and the data of 50 images were analyzed.

### Measurement of average PDFF from whole pancreas by three-dimensional analysis

**Fig 1:**
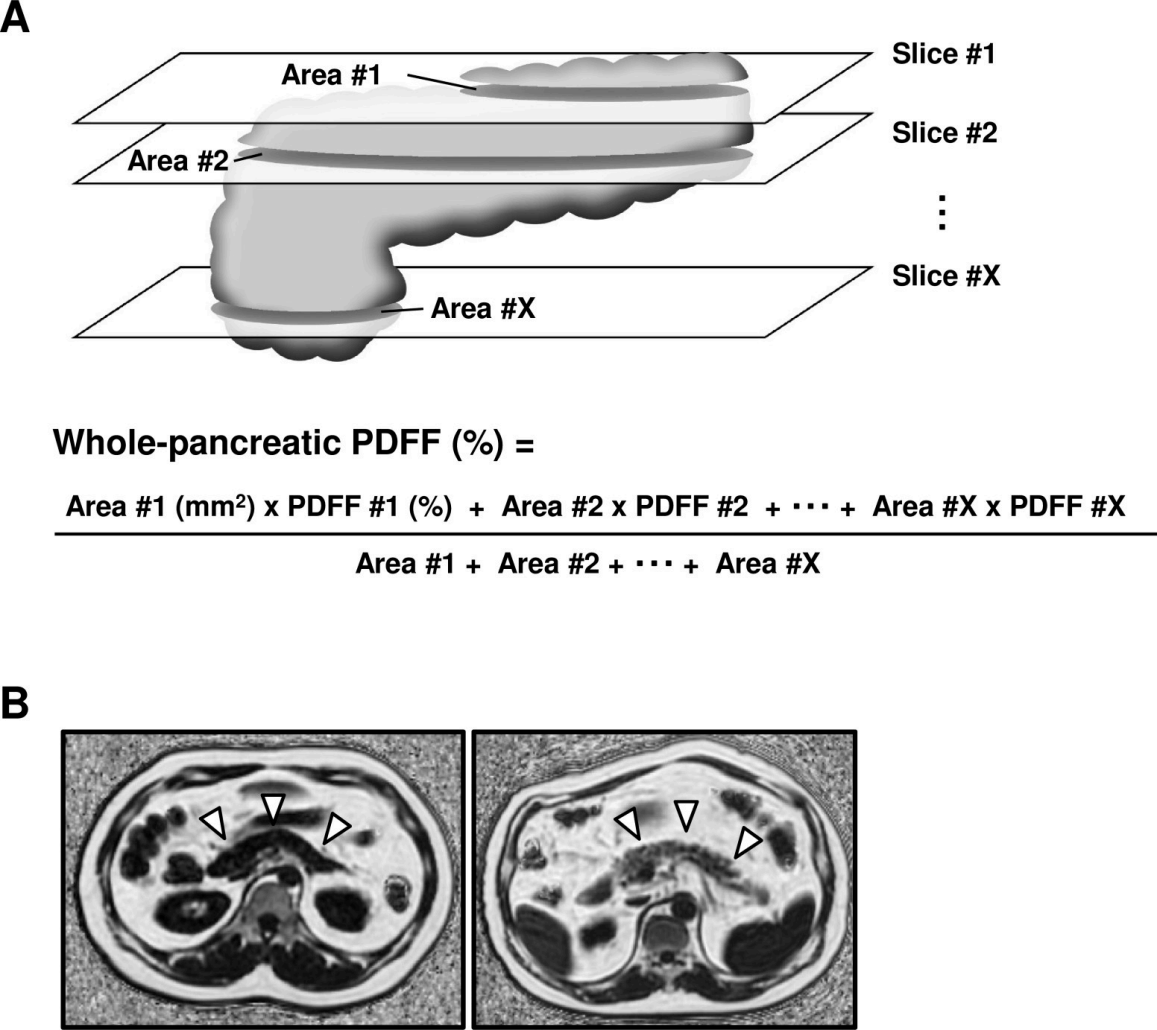
The schema of whole-pancreatic analysis and representative fat-water MRI images of low or high degree of pancreatic steatosis. (A) The schema of whole-pancreatic analysis is shown. (B) Representative pancreatic images of fat-water MRI are shown. A white arrow indicates the pancreas. The left panel shows the low degree of pancreatic steatosis in a sample whose whole-pancreatic FF value was determined to be 1.20%. The right panel shows the high degree of pancreatic steatosis in a sample whose whole-pancreatic FF value was determined to be 34.87%.

Using the free-hand ROI tool, we drew the ROI in the area of the pancreas appearing in each slice. On each slice, the area of the ROI and the average PDFF of the voxels included in the ROI were calculated by the IDEAL-IQ software. After drawing the ROIs in all the slices in which the pancreas appeared, we calculated the average FF of all the voxels included in the pancreas by the following formula; [Area #1 x PDFF #1 + Area #2 x PDFF #2 + … + Area #X x PDFF #X] / (Area #1 + Area #2 + … + Area #X) x 100%. We named this average PDFF “whole-pancreatic PDFF.” The average value of three observers was taken as the measured value. The schema of whole-pancreatic analysis is shown in Fig 1A and representative images of low and high whole-pancreatic PDFF values are shown in Fig 1B.

### Evaluation of the intra-observer and inter-observer variability of the new measurement method

Seven observers (S.K., A.I., Y.K., J.A., K.I., M.Y., and T.Y.) measured 50 images independently. Each observer measured each image five times on different days. Of the seven, six are gastroenterologists and one is a radiologist. Two of the liver experts are authors of the previously reported MRI analysis [6]. The intra-observer variability of each observer was analyzed by calculating the coefficient of variation (CV) of 5 measurements in each image. The inter-observer variability was assessed by calculating the CV of 7 measurements in each image by 7 observers.

### Measurement of average PDFF from a part of pancreas by three-dimensional analysis

We analyzed the differences in the degree of steatosis among different regions of the pancreas. Using the free-hand ROI tool, we drew the ROI in the area of the pancreas head alone, as well as that of the pancreatic body and tail alone. Then, we calculated the PDFF of the pancreatic head alone (hereinafter referred to as head-PDFF), as well as that of the pancreatic body and tail alone (hereinafter referred to as body-tail PDFF), in the same way as whole-pancreatic PDFF. These two parts of the pancreas were divided at the location of the portal vein.

### Statistical analysis

All the statistical analyses were performed using the SPSS software (version 12, SPSS Inc., Chicago, IL). Mann-Whitney’s U-test was used for univariate comparisons among groups. The Kruskal-Wallis test was used for comparisons of more than two independent groups. Multiple logistic regression analysis was then carried out using the following factors as the covariates: age, sex, BMI, presence/absence of diabetes, serum triglyceride (TG), LDL-C and LH ratio (ratio of serum low-density lipoprotein cholesterol to serum high-density lipoprotein cholesterol). The multivariate model calibration was assessed by the Hosmer-Lemeshow test of goodness-of-fit. Spearman’s rank correlation coefficient was used for analyzing the correlation between the hepatic PDFF and pancreatic PDFF. In this study, *p* < 0.05 was considered indicative of statistical significance. All the authors had full access to all the study data and were involved in the review and approval of the final manuscript.

## Results

### Pancreatic PDFF showed large variations depending on the locations of the ROIs

**Fig 2:**
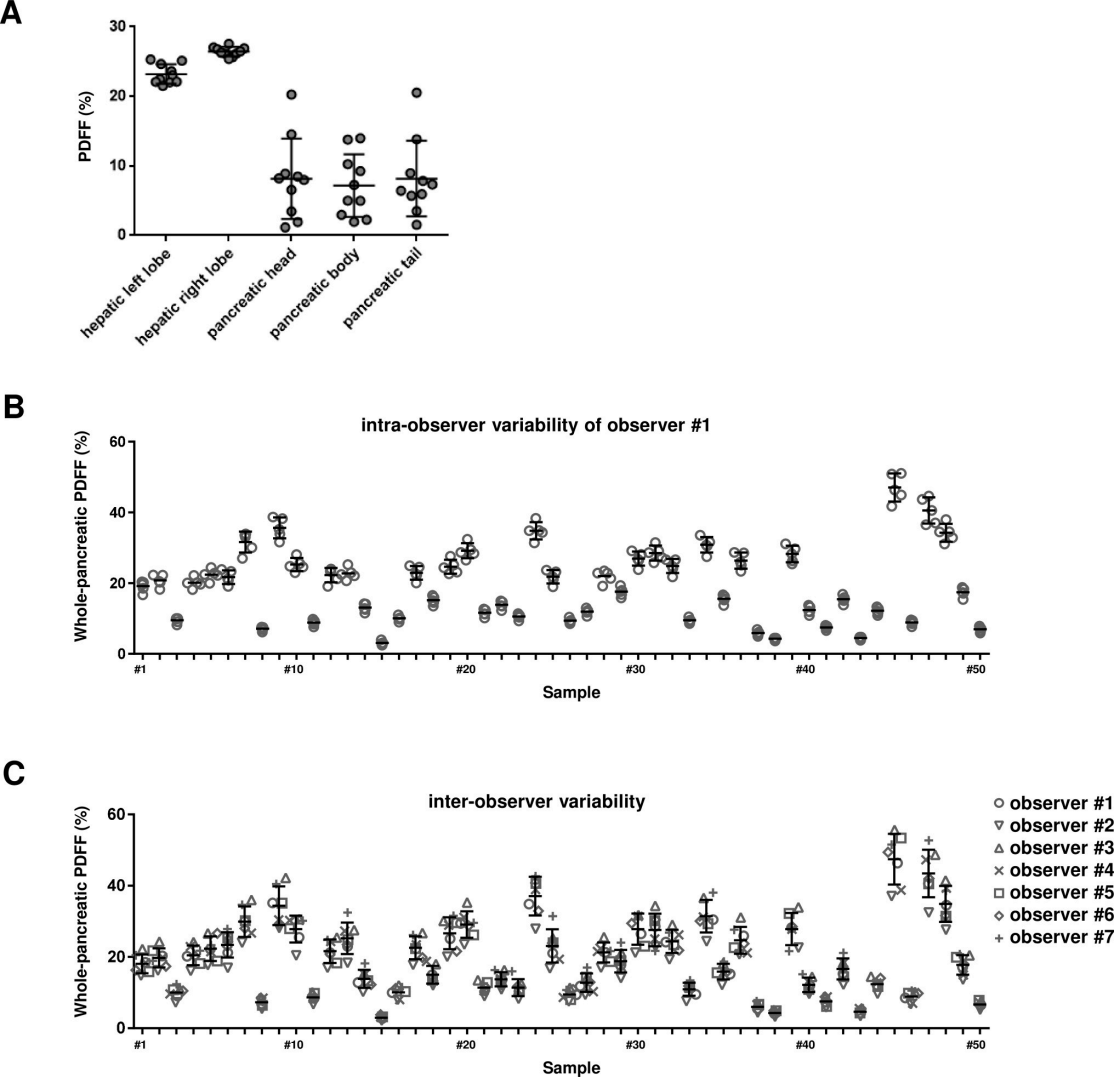
Evaluation of reproducibility of each measurement method. (A) The representative result of the evaluation of the variations in values of hepatic or pancreatic PDFF depending on the positions of the ROIs is shown. The values of the pancreatic PDFF measured using small ROIs varied widely depending on the locations of the ROIs. (B) All measurements by observer #1 for intra-observer variability analysis are shown. (C) All measurements by 6 observers for inter-observer variability analysis are shown. The average value of five repeated measurements by each observer was taken as the measured value of the observer.

Firstly, using the MRI images of patients with NAFLD, we evaluated the variations in values of hepatic and pancreatic PDFF depending on the positions of the ROIs. A total of 50 images were analyzed, and the representative result is shown in Fig 2A. There were no significant variations in the results among the images. Measurement of the PDFF using ROIs set in multiple different positions in the liver and pancreas demonstrated that the variations in values depending on the positions of the ROIs were significantly greater for the pancreatic PDFF than for the hepatic PDFF. The mean coefficients of variation (CV) of the PDFF values among ten ROIs in the hepatic left robe and right lobe were 6.71% and 6.10%, respectively. The mean CVs those in the pancreatic head, body and tail were 61.0%, 64.2% and 69.2%, respectively.

### Evaluation of intra-observer and inter-observer variability of the new measuring method

Next, we evaluated the intra-observer and inter-observer variability in the measured values of our three-dimensional analysis (Fig 1A). Seven observers measured 50 images independently. Each observer measured each image five times. All measurements by observer #1 for intra-observer variability analysis are shown in Fig 2B. The mean CV of 5 measurements in 50 images was 8.36% in observer #1. This mean CV did not change significantly in the other 6 observers, all less than 10% (6.71%, 6.47%, 6.92%, 8.04%, 6.72%, and 8.12%). All measurements by 7 observers for inter-observer variability analysis are shown in Fig 2C. The mean CV of 7 measurements by different observers in 50 images was 11.39%.

### Whole-pancreatic PDFF was correlated with age, BMI, and presence/absence of diabetes

**Fig 3:**
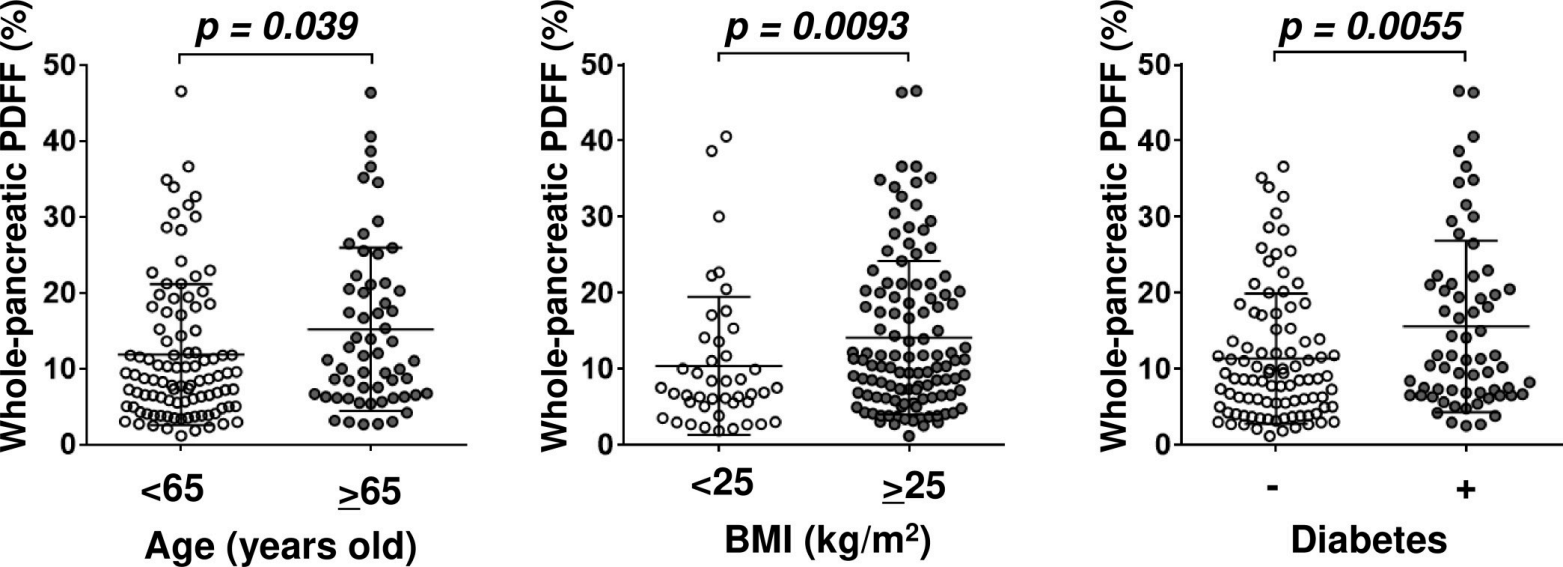
Correlation between whole-pancreatic PDFF and the clinical characteristics. Whole-pancreatic FF was correlated with age (left panel, *p* = 0.039), BMI (middle panel, *p* = 0.0093), and presence/absence of diabetes (right panel, *p* = 0.0055).

We analyzed whole-pancreatic PDFF determined by our three-dimensional analysis and clinical data of 159 patients with NAFLD, whose clinical characteristics are shown in Table 1. The results revealed significant correlations of the whole-pancreatic PDFF with age, body mass index (BMI), and presence/absence of diabetes (Fig 3). We also evaluated the correlations between the whole-pancreatic PDFF and other clinical characteristics. As shown in Table 2, there were no significant linear correlations between the whole-pancreatic PDFF and any of the other clinical characteristics examined except Hemoglobin A1c (HbA1c).

**Table 1:** Clinical, serologic and histologic characteristics of the subjects.

Characteristic	
N	159
Age, y, mean ± SD	57.2 ± 14.5
Sex, male/female	87 / 71
BMI, kg/m^2^, mean ± SD	28.1 ± 4.6
Diabetes, %	41.1
HbA1c, %, mean ± SD	6.3 ± 1.1
Fasting Blood Glucose, mg/dl, mean ± SD	122.0 ± 40.5
HOMA-IR (n = 129), mean ± SD	7.4 ± 11.2
smoke, %	32.3
Hypertension, %	41.1
Dyslipidemia, %	79.1
TG, mg/dl, mean ± SD	171 ± 127
LDL-C, mg/dl, mean ± SD	118.2 ± 31.1
HDL-C, mg/dl, mean ± SD	55.5 ± 15.7
LH ratio, mean ± SD	2.29 ± 0.85

**Table 2:** Correlation between whole-pancreatic PDFF and the clinical characteristics.

	Pancreas			Liver
	Whole	Head	Body and Tail	
Characteristics	*p* value	*p* value	*p* value	*p* value
Sex	0.44	0.58	0.28	0.91
Age >65 y	0.039*	0.49	0.0063*	<0.0001*
BMI >25 kg/m^2^	0.0093*	0.0011*	0.058	0.019*
Diabetes	0.0055*	0.0035*	0.0149*	0.16
HbA1c >6.5%	0.0010*	0.00050*	0.0044*	0.43
Fasting Blood Glucose >126mg/dl	0.28	0.20	0.36	0.082
HOMA-IR >2.5**	0.67	0.70	0.77	0.069
Smoker	0.75	0.15	0.31	0.69
Hypertension	0.070	0.053	0.15	0.73
Dyslipidemia	0.50	0.61	0.15	0.053
TG >150 mg/dl	0.46	0.17	0.78	0.0059*
LDL-C >140 mg/dl	0.071	0.027***	0.25	0.26
LH ratio <1.5	0.15	0.22	0.12	0.016*

*Significant

**For analysis of HOMA-IR, the sample number decreased to n = 129 because of missing data.

***Inverse correlation

### Correlations with the clinical characteristics differed depending on the location of measurement of the PDFF in the pancreas

As pancreatic steatosis is reported to be patchy in nature [12], we wanted to know whether the clinical characteristics related to pancreatic steatosis differ depending on the location in the pancreas. We analyzed the correlations between the pancreatic PDFF measured in different locations of the pancreas and the clinical characteristics, and found some noteworthy differences depending on the location of measurement in the pancreas. Interestingly, although the presence/absence of diabetes and HbA1c were correlated with both the head-PDFF and body-tail PDFF, age was correlated with only the body-tail PDFF, and BMI was correlated with only the head-PDFF (Table 2). Moreover, to our surprise, the serum level of low-density lipoprotein cholesterol (LDL-C) was inversely correlated with the head-PDFF (Table 2).

### Risk factors for pancreatic steatosis

To examine the significant risk factors for pancreatic steatosis, we performed a multivariate analysis including the clinical characteristics as covariates. Multiple logistic regression analysis identified elevated BMI, being male, advanced age, and presence of diabetes as being positively associated with the values of the whole-pancreatic PDFF (Table 3). Elevated BMI, presence of diabetes, and elevated TG were positively associated with the values of the head-FF; elevated LDL was negatively associated with the values of the head-PDFF (Table 3). Advanced age, elevated BMI, and being male were positively associated with the values of the body-tail PDFF (Table 3).

**Table 3:** Identified factors which were correlated with the degree of pancreatic steatosis by multiple logistic regression analysis.

**Whole-pancreatic PDFF**			
	**Factors**	***p* value**	**OR (95% CI)**
	BMI >25 kg/m^2^	0.00095	4.13 (1.78–9.58)
	Sex (male/female)	0.0015	3.21 (1.57–6.57)
	Age >65 y	0.0065	2.94 (1.35–6.37)
	Diabetes (presence/absence)	0.0041	2.06 (1.03–4.14)
**Head-PDFF**			
	**Factors**	***p* value**	**OR (95% CI)**
	BMI >25 kg/m^2^	0.0045	3.14 (1.42–6.90)
	Diabetes (presence/absence)	0.014	2.44 (1.20–4.97)
	TG >150 mg/dl	0.042	2.06 (1.03–4.11)
	LDL >140 mg/dl	0.0065	0.31 (0.14–0.72)
**Body-tail PDFF**			
	**Factors**	***p* value**	**OR (95% CI)**
	Age >65 y	0.00021	4.48 (2.02–9.89)
	BMI >25 kg/m^2^	0.020	3.76 (1.62–8.73)
	Sex (male/female)	0.033	2.14 (1.06–4.31)

Hosmer-Lemeshow Test: *p* = 0.87 (whole-pancreatic PDFF), *p* = 0.51 (Head-PDFF), *p* = 0.61 (Body-tail PDFF).

### Whole-pancreatic PDFF was not correlated with the hepatic PDFF or NAS score

**Fig 4:**
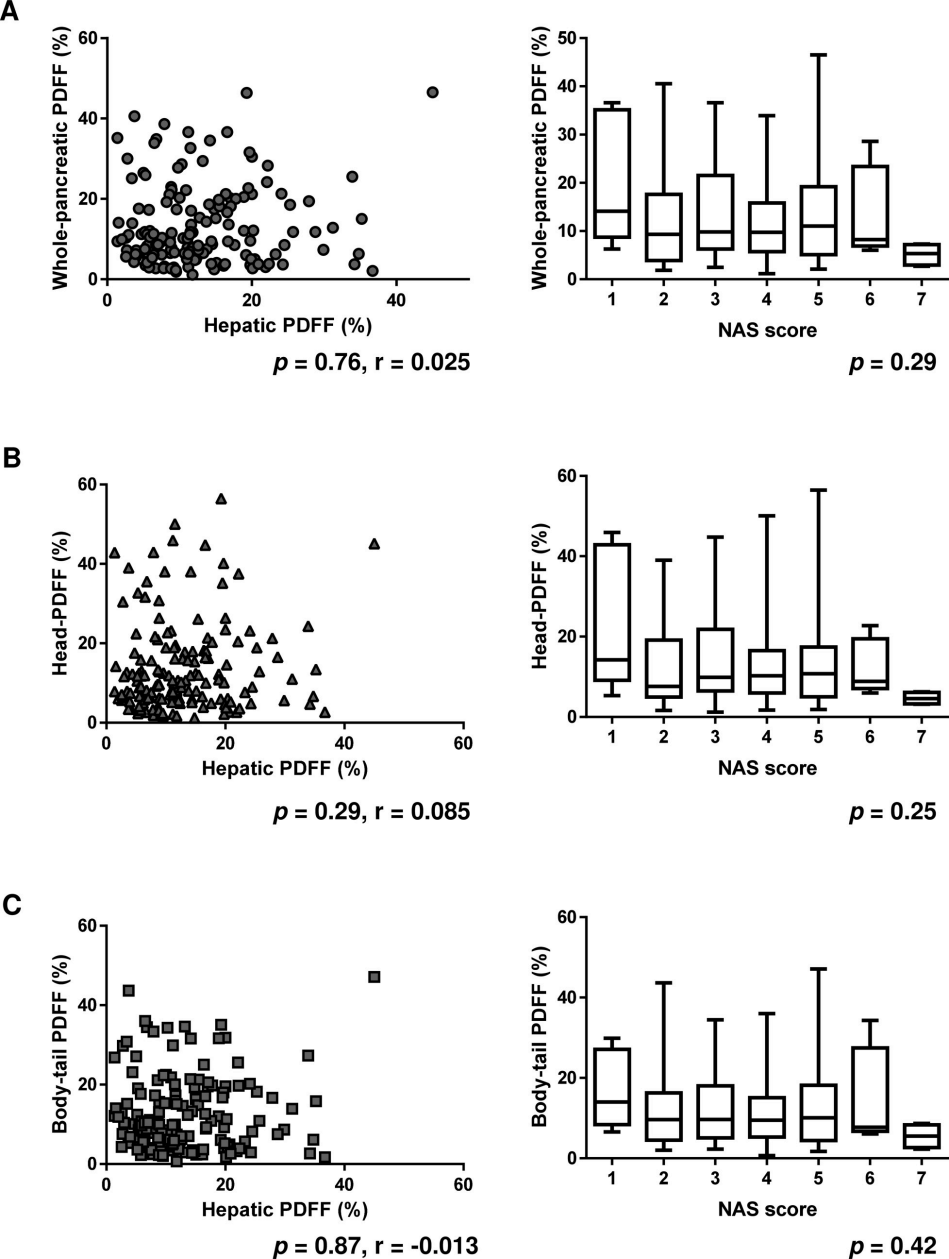
Correlation between whole-pancreatic PDFF and the hepatic PDFF or NAS score. Whole-pancreatic PDFF was not correlated with hepatic PDFF (left panel, *p* = 0.76), and NAS score (right panel, *p* = 0.29). (B) Head-PDFF was not correlated with hepatic PDFF (left panel, *p* = 0.29), and NAS score (right panel, *p* = 0.25). (C) Body-tail PDFF was not correlated with hepatic PDFF (left panel, *p* = 0.87), and NAS score (right panel, *p* = 0.42).

Finally, we attempted to determine the correlation between hepatic and pancreatic steatosis. We compared the pancreatic PDFF to the hepatic PDFF and non-alcoholic steatohepatitis activity score (NAS score). The results revealed that the whole-pancreatic PDFF was not correlated with either the hepatic PDFF (*p* = 0.76) or the NAS score (*p* = 0.29) (Fig 4A). We also compared the associations of the head-PDFF and body-tail PDFF with the hepatic PDFF and NAS scores, and found that neither was correlated with the hepatic PDFF (*p* = 0.29, *p* = 0.87, respectively) or NAS scores (*p* = 0.25, *p* = 0.42, respectively) (Fig 4B and 4C). Analysis of the correlations between the hepatic PDFF and clinical features demonstrated that the hepatic PDFF was mainly correlated with dyslipidemia-related features, such as the serum TG (*p* = 0.0059), LDL-C and LH ratio (*p* = 0.016) (Table 2).

## Discussion

In this study, we firstly showed the large variations of pancreatic PDFF depending on the locations of the ROIs when we used ROIs with a small area. This finding suggests that the pancreatic PDFF determined by the measuring method based on the small ROIs may not represent the degree of pancreatic steatosis. To determine the representative value of pancreatic PDFF, we developed a new measurement method for pancreatic PDFF, which calculated the average PDFF from all the voxels included in the pancreas. The value of pancreatic PDFF calculated by our method, which is called whole-pancreatic PDFF, demonstrated greater repeatability and reproducibility of the measured values. Analysis of correlations with clinical characteristics showed that whole-pancreatic PDFF was correlated with age, BMI and presence/absence of diabetes. Analysis based on measurement of the head-FF and body-tail PDFF separately revealed that different clinical characteristics correlated with the two areas. Multivariate analysis identified some risk factors for pancreatic steatosis. Finally, we found that the whole-pancreatic PDFF was not correlated with the hepatic PDFF value or NAS score.

Firstly, we showed great variability of the pancreatic PDFF depending on the locations of the ROIs when we used ROIs with a small area (Fig 2A). Such large variability of the values depending on the ROIs selected reflects, as reported previously, the patchy nature of pancreatic steatosis [12], and that the PDFF determined from ROIs set in only a part of the pancreas is not representative of the degree of steatosis in the entire pancreas. Moreover, such large variability of measurement results causes a great inter-examiner variability of the determined pancreatic PDFF values. Considering the patchy distribution of steatosis in the pancreas, the entire pancreas should be considered in any discussion of the severity of pancreatic steatosis.

Our three-dimensional measurement method allows the analysis of the average PDFF of the entire pancreas. This average PDFF is thought to be more representative of the degree of pancreatic steatosis than PDFF determined by multiple small ROIs. Additionally, the intra- and inter-examiner variability of the measurement results of our method was acceptable as compared to the much higher CV of the values measured using small ROIs (head; 61.0%, body; 64.2%, tail; 69.2%) (Fig 2B). In quantitative analysis, repeatability and reproducibility are important factors determining the usefulness of the analysis. Thus, our method could be useful for the detailed characterization of pancreatic steatosis.

Univariate analysis demonstrated that whole-pancreatic PDFF was correlated with diabetes-related characteristics rather than dyslipidemia, such as HbA1c (Table 2). One of the advantages of our new measurement method is that it enables a comprehensive analysis of steatosis in any three-dimensional spaces of interest. Taking advantage of this feature, we measured the head-FF and body-tail PDFF separately for the detailed characterization of pancreatic steatosis. Univariate analysis revealed that age is correlated with only body-tail PDFF, and BMI is correlated with only head-PDFF (Table 2). This difference of related clinical characteristics between head-PDFF and body-tail PDFF was also demonstrated by multivariate analysis (Table 3). Considering that the pancreas is a composite organ derived from two buds, dorsal and ventral [14], the mechanism of steatosis might differ depending upon the location of the fat accumulation in the pancreas. These findings also support the idea that for the quantification of pancreatic steatosis, the entire pancreas should be analyzed.

The inverse correlation between the serum LDL-C level and the head-PDFF is a novel finding in this study. Elevated serum LDL-C is reported as a risk factor for multiple diseases, such as cardiovascular disease [15], ischemic stroke [16], and NAFLD [17]. Thus, measures to decrease the serum LDL-C level are generally recommended. However, our results indicate that maintenance of the elevated serum LDL-C might prevent steatosis of the pancreatic head and that excessive decrease of LDL-C might promote steatosis of the pancreatic head and the consequent development of diabetes. Although further analysis is necessary to conclude the relationship between the serum LDL-C level and pancreatic steatosis, these findings are expected to contribute to the determination of the appropriate target LDL-C level in patients receiving drugs for dyslipidemia.

Finally, we showed the absence of any significant correlation between hepatic and pancreatic steatosis. Although some authors have demonstrated a correlation between hepatic steatosis and pancreatic steatosis using different modalities [9, 18, 19], our results did not show any correlation between the two. In fatty liver, fat is observed inside the hepatocytes, causing “ballooning” of the hepatocytes [20]. In the case of pancreatic steatosis, adipocyte infiltration is observed outside the acinar cells [21]. These findings suggest that the mechanism of steatosis may also differ between the pancreas and liver.

One of the important limitations of this study is the lack of histological samples. Since they were not available, we couldn’t directly compare the histological severity of pancreatic steatosis and the value of PDFF in this study. However, even though surgically resected or needle biopsy samples are available, it is difficult to analyze the entire pancreas with them. Thus, the analysis with autopsy samples is helpful to characterize the histological severity of the whole pancreas in that the entire pancreas can be analyzed. The previous reports demonstrated that the severity of histological pancreatic steatosis in autopsy samples was correlated with the degree of obesity [3], age [3] and presence/absence of diabetes [3, 4]. These three clinical characteristics were also correlated with whole-pancreatic PDFF in our analysis. Thus, although further analysis with histological samples is required to determine reference values for PDFF that defines pancreatic steatosis, whole-pancreatic FF is considered to be reliable as a value that reflects the severity of steatosis of the whole pancreas.

Another limitation is the lack of healthy control samples. Even if MRI is a non-invasive examination, ethical validity is essential to obtain images of healthy people. Thus, healthy control samples are not included in this initial study. Further analysis in healthy controls is needed to determine the exact normal range of whole-pancreatic PDFF values.

In conclusion, we developed a new measurement method of fat-water MRI for pancreatic steatosis. Our three-dimensional measurement method allows the analysis of the entire pancreas with increased repeatability and reproducibility of the measured values. The reduced variability of the measured values would help in obtaining reproducible analysis results and detailed characterization of pancreatic steatosis.
